# Comparing the Colloidal Stabilities of Commercial and Biogenic Iron Oxide Nanoparticles That Have Potential In Vitro/In Vivo Applications

**DOI:** 10.3390/molecules28134895

**Published:** 2023-06-21

**Authors:** Jonas Schwan, Simon Markert, Sabine Rosenfeldt, Dirk Schüler, Frank Mickoleit, Anna S. Schenk

**Affiliations:** 1 Physical Chemistry IV, University of Bayreuth, D-95447 Bayreuth, Germany; 2 Department Microbiology, University of Bayreuth, D-95447 Bayreuth, Germany; 3 Physical Chemistry I, University of Bayreuth, D-95447 Bayreuth, Germany; 4 Bavarian Polymer Institute (BPI), University of Bayreuth, D-95447 Bayreuth, Germany

**Keywords:** magnetic nanoparticles, magnetosomes, colloidal stability, protein corona

## Abstract

For the potential in vitro/in vivo applications of magnetic iron oxide nanoparticles, their stability in different physiological fluids has to be ensured. This important prerequisite includes the preservation of the particles’ stability during the envisaged application and, consequently, their invariance with respect to the transfer from storage conditions to cell culture media or even bodily fluids. Here, we investigate the colloidal stabilities of commercial nanoparticles with different coatings as a model system for biogenic iron oxide nanoparticles (magnetosomes) isolated from magnetotactic bacteria. We demonstrate that the stability can be evaluated and quantified by determining the intensity-weighted average of the particle sizes (*Z*-value) obtained from dynamic light scattering experiments as a simple quality criterion, which can also be used as an indicator for protein corona formation.

## 1. Introduction

As they offer a large number of possible applications and can be used with high flexibility, for example as drug carriers, as agents for magnetic imaging or in the treatment of tumor cells in the context of magnetic hyperthermia, magnetic iron oxide nanoparticles are increasingly attracting interest for biomedical and clinical applications [1,2,3,4]. However, meeting the requirements for biocompatible nanoparticle (NP) formulations, specifically the colloidal stability, desired magnetic properties and uniform size and shape, remains challenging. Despite remarkable progress in the field, particle synthesis and scaled-up production still need to be optimized. Additionally, key issues, such as the biocompatibility, have to be adequately addressed [5]. Most iron oxide NPs are produced by chemical synthesis routes. A promising method for scalability and process control is the hydrothermal synthesis; however, this requires high reaction temperatures [6]. Other methods include sol-gel synthesis or liquid and gas phase methods. Generally, irrespective of the synthesis route, coatings are necessary to prevent aggregation of the particles and thus provide colloidal stability as well as biocompatibility. However, the controlled formation of effective ligand shells for the magnetic nanoparticles is still a major challenge [5].

Magnetosomes, i.e., biogenic magnetic nanoparticles (b-NPs) synthesized by magnetotactic bacteria, offer a promising alternative to artificially produced nanoparticles since they are naturally membrane enveloped and formed in a genetically strictly controlled biomineralization process. For example, the alphaproteobacterium *Magnetospirillum gryphiswaldense* synthesizes cuboctahedral, nanometer-sized (35–40 nm in diameter) magnetic crystals of chemically pure magnetite (Fe_3_O_4_) enveloped by a proteinaceous phospholipid bilayer [7]. The latter not only has a crucial function in the mechanism of biomineralization but also contributes to the colloidal stabilization of the nanoparticles [8,9,10]. Furthermore, the membrane and its embedded proteins provide sites for the covalent attachment of further protein cargo, enabling the decoration of the particle surface with functionalities such as fluorophores, enzymes, coupling groups or artificial peptides to tune the particles’ surface characteristics [11,12,13]. Thereby, in particular the in vivo functionalization by genetic engineering enables the controllable and highly selective display of foreign proteins at distinct stoichiometries, expressed as translational fusions to abundant magnetosome membrane proteins [14].

In summary, magnetosomes exhibit very valuable characteristics due to the fully genetically controlled biomineralization process by which they are formed, including high crystallinity, narrow particle size distribution and strong magnetization, endowing them with various advantages over chemically synthesized nanoparticles in bio-related applications [15,16,17,18]. Recent studies have shown that magnetosomes from *M. gryphiswaldense* can be easily isolated with high purity by magnetic separation techniques and are considered biocompatible even at increased particle concentrations (i.e., the viability of mammalian cell lines is only slightly affected) [17,19,20].

For most future biomedical and clinical medical applications, however, it has to be ensured that the aggregation of the nanoparticles in suspension is prevented. In such applications, the diameters of the individual particles, potentially formed clusters or agglomerates are crucial for their biodistribution, and these parameters are directly related to the particle clearance from the blood stream. Particles in the size range <200 nm have slower removal rates compared with larger ones (>200 nm) due to fast uptake by the mono-nuclear phagocyte system. Whereas smaller particles <10 nm are removed from the body by renal clearance, nanoparticles >100 nm are eliminated by macrophages, mostly after accumulation in the liver and spleen [3,4]. Nanoparticles with diameters between 10 nm and 100 nm were reported to be well-suited for successful clinical application. Thereby, ~50 nm particles or nanoconjugates (aggregates or clusters) could be close to the optimal size for overall tumour tissue accumulation and retention.

A suitable coating of nanoparticles can (i) prevent cluster formation, (ii) enables functionalization of the particle surface through the attachment of suitable (bio)molecules and (iii) at the same time can prevent the adsorption of blood plasma proteins, a complication that can lead to faster degradation and accumulation in the organs of the reticuloendothelial system (a system of connective tissues that are part of the immune system) [21,22]. Thus, the cytotoxicity and biocompatibility of the magnetic nanoparticles generally strongly depend on the coating (and the surrounding medium) of the iron oxide cores and less on the cores themselves [23].

**Figure 1:**
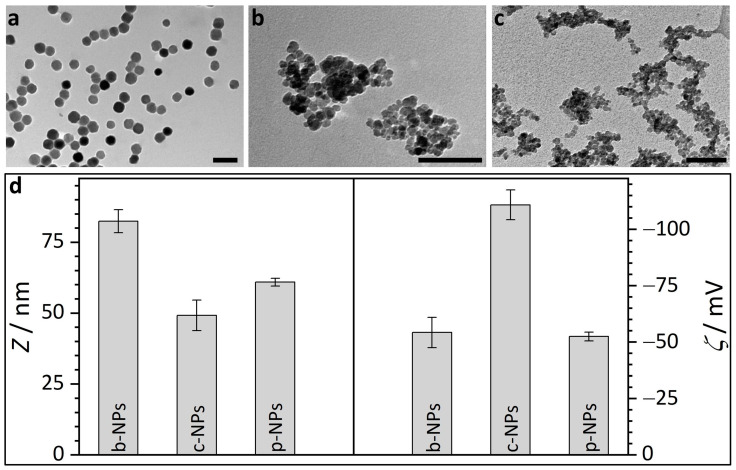
Transmission electron micrographs and DLS results for different types of iron oxide nanoparticles. (**a**) TEM image of biogenic magnetosomes isolated from *M. gryphiswaldense* (b-NPs). (**b**) TEM image of commercial, citric acid encapsulated iron oxide nanoparticles (c-NPs). (**c**) TEM image of commercial phospholipid encapsulated iron oxide nanoparticles (p-NPs). Scale bar for TEM micrographs (**a**–**c**): 100 nm; scale bar insets: 50 nm. (**d**) Average diameter *d* and zeta potential *ζ* for the different iron oxide nanoparticle suspensions in water.

In the work presented here, we address the stability of suspensions of different types of iron oxide nanoparticles (i.e., biogenic or commercial and chemically synthesized), which are strongly dependent on the conditions and media to which they are exposed. In detail, we assess bacterial magnetosomes (b-NPs) isolated from *M. gryphiswaldense* and chemically synthesized iron oxide core nanoparticles stabilized by a citric acid (c-NPs) or phospholipid (p-NPs) matrix (Figure 1). Specifically, we focus on commonly used buffer solutions, such as 4-(2-hydroxyethyl)-1-piperazineethanesulfonic acid (HEPES), as well as two different commonly used cell culture media, namely “Roswell Park Memorial Institute” (RPMI) medium and “Dulbecco’s Modified Eagle Medium” (DMEM). As both contain a variety of different compounds with a not exactly defined composition (e.g., fetal bovine serum, FBS), potential particle–particle interactions are investigated with regard to influences on the colloidal stability. The newly gained knowledge might contribute to a better understanding of the stabilization of suspensions of (biogenic) magnetic nanoparticles in order to take a next step towards potential in vitro/in vivo applications.

## 2. Results and Discussion

### 2.1. Size and Size Distribution of Iron Oxide Core Nanoparticles

A coherent study of the nanoparticle stability in various media requires a precise picture of NP size and the size distribution. Hence, the size of different iron oxide nanoparticle types was examined by applying a complementary set of techniques including transmission electron microscopy (TEM), dynamic light scattering (DLS) and small angle X-ray scattering (SAXS). To correctly interpret the data, it is important to note that the NPs consist of a core–shell structure. An overview of the iron oxide size values determined from these measurements is given in the Supporting Information (Table S1).

The investigated biogenic nanoparticles (b-NPs) consist of magnetic (single-domain) nanocrystals with a cuboctahedral magnetite (Fe_3_O_4_) core. It has been demonstrated by means of synchrotron X-ray diffraction that intracellular magnetite is structurally pure, suggesting that the bacteria generate optimal physicochemical conditions to protect the core against oxidation [24]. The core itself is surrounded by a phospholipid bilayer (magnetosome membrane), which harbors a set of magnetosome-specific proteins (Figure S1). Magnetosomes can be isolated with intact membranes; however, the latter cannot fully prevent oxidation of the magnetite cores of purified particle suspensions. Thus, even under storage conditions (HEPES buffer at 4 °C under a nitrogen atmosphere), a drop of the saturation magnetization to one-third of its initial value was observed over a period of one year [25].

Transmission electron micrographs, in principle, provide an overview over the morphology of the biogenic core-shell nanoparticles; however, due to the high electron density contrast difference between magnetite and the magnetosome membrane (ca. 1500 electrons/nm^3^ for magnetite vs. ca. 650 electrons/nm^3^ for the membrane according to gray scale analysis) even after staining, commonly only the magnetosome core is addressed. The biogenic iron oxide nanoparticles from *M. gryphiswaldense* (Figure 1a) show uniform size and shape. TEM image analysis reveals a magnetosome size of about 40 ± 2 nm (Figure 1a). In TEM evaluation, however, the shell size, i.e., the thickness of the magnetosome membrane, proved difficult to estimate. Although the general contrast situation is similar to TEM, SAXS as a volume-averaging, quantitative technique allows for the estimation of the maximal membrane thickness in highly concentrated or sedimented samples, as shown in a previous work [26]. SAXS analysis based on a model of (interacting) polydisperse spheres revealed a diameter of 32.0 ± 5.4 nm for the isometric iron oxide core, whereas the thickness of the magnetosome membrane was determined to be <6 nm [26]. However, SAXS experiments are commonly restricted to concentrated suspensions (ca. >1 wt%) that are far above the concentration regime required for most practical applications (clinical doses are usually in the range between 0.6 and 8 mg iron species (Fe) per kg patient body weight [27,28]). Further, the size of the nanoparticles, including their (solvent-swollen) biological membrane as well as their tendency towards aggregation, strongly depends on the charge/ion concentrations around the particles, i.e., the overall size of magnetosomes in water will certainly differ from the dimensions in a high-salt medium. For lower concentrations (ca. <1 wt%), DLS provides a simple, cost-efficient, commercially available and omnipresent analytic tool to characterize the hydrodynamic radii and thus the particle or cluster sizes in solution. Therefore, in this study DLS measurements were performed on aqueous particle suspensions.

Here, biogenic magnetosomes from *M. gryphiswaldense* (b-NPs) exhibited an average size of *d* = 83.5 ± 2.8 nm (Figure 1d, Table S2). These values agree with previous studies, in which hydrodynamic diameters of ca. 76 nm were reported [28]. The difference between the size parameters determined from TEM images (ca. 40 nm, magnetite crystal only), SAXS (ca. 32 nm iron oxide core, membrane thickness <6 nm) and the DLS results (ca. 84 nm, magnetosome size) may indicate that the biological membrane of the magnetosomes in water swells up to an unknown degree. However, it cannot be excluded that the sizes obtained from the DLS measurements represent an average value of a high amount of small individual particles and few aggregates.

The magnetic properties of magnetosomes (b-NPs) have been investigated in various studies [25,29,30,31,32,33]. Due to their particle diameters, b-NPs are near the transition range between superparamagnetic and ferrimagnetic/stable single-domain behavior (at ~30 nm). Thereby, the co-existence of stable single-domain and superparamagnetic particles has been reported. For freshly isolated b-NPs the saturation magnetization is in the range of 70–110 A m^2^ kg^−1^.

The magnitude of repulsion/attraction interactions between particles is one of the fundamental parameters affecting colloidal particle stability. When the charge drops below a specific value, the colloids start to flocculate, conjugate or sediment due to the lack of repulsive forces. The electrostatic properties of the particle surfaces can be described by the zeta potential *ζ* (voltage at the slipping plane where the diffuse counterion layer of the colloid meets the surrounding liquid), which can be measured in principle by DLS but on practical grounds is often restricted to suspensions/solutions with low ionic strength to prevent undesired reactions between ions from the sample solution and the electrodes of the measurement cell. The higher the magnitude of ζ, the stronger the electrostatic repulsion between single particles and the more likely the requirements for a stable suspension are fulfilled. A suspension with a zeta potential value of ζ > |30| mV is usually considered as stable. Accordingly, the zeta potential of *ζ* = −54.2 ± 6.7 mV measured for biogenic magnetosomes (Figure 1d, b-NPs) in water, reveals the pronounced stabilization capacity of the magnetosome membrane. The *ζ* values for b-NPs are more negative than the published values for pure Fe_3_O_4_ NPs and ethylenediaminetetraacetic acid (EDTA)-coated iron oxide NPs, which are in the range of −30 mV [34]. *ζ* values similar to b-NPs are published for sodium dodecyl sulfate (SDS)-coated Fe_3_O_4_ NPs (ca. −50 mV) [35].

Commercial iron oxide nanoparticles with a citric acid ligand shell for stabilization (c-NPs) are present as aggregates/clusters of individual particles, as obvious from the TEM images (Figure 1b). Hence, the determination of the size of individual particles as well as the averaged cluster size are uncertain and prone to errors. However, values roughly in the range of 10 nm (averaged individual unit) and 60 nm (mean cluster size) are obtained. The datasheet provided by the supplier (chemicell GmbH) describes the iron oxides in aqueous suspension as superparamagnetic and suitable for MRI (magnetic resonance imaging) diagnostics or for binding of cationic molecules. However, due to the fact that the c-NPs are multi-core clusters consisting of cohesive nanoparticles, the magnetic susceptibility of the c-NPs may be influenced by the internal collective organization of the individual superparamagnetic units. Cabrera et al. investigated the magnetic behavior of c-NPs (same supplier/product number as in our study) dispersed in double-distilled water (ddH_2_O) [36]. The authors reported the coexistence of Brownian and Néel relaxation, a distinct hysteretic behavior and a specific absorption rate (SAR) of 40 ± 2 W g^−1^. Citric-acid-stabilized iron oxide nanoparticles with a core size of approx. 10 nm were chemically synthesized by Lartigue et al. via the oxidation of magnetite [37]. The particles exhibited a saturation magnetization being diminished by ca. 30% compared with the value of bulk maghemite (bulk: 80 A m^2^ kg^−1^). For c-NPs, the supplier provides three hydrodynamic diameters, ~50 nm, ~100 nm and ~200 nm, for fractions of 87%, 12% and 1% of the particles, respectively. In our study, the citric-acid-coated nanoparticles were additionally analyzed by DLS and show an average diameter of *d* = 59.3 ± 7.2 nm and a *ζ* potential of −110.8 ± 6.6 mV (Figure 1d). In comparison with b-NPs, the smaller size and the higher value of *ζ* for the c-NPs would theoretically point to more stable NPs. However, the aggregation clearly visible in the TEM images indicates the contrary, i.e., the DLS-derived values do not belong to the individual c-NP units but represent the averaged dimensions/properties of the clusters. A possible explanation for the strong tendency towards aggregation might be the influence of the attractive magnetic interactions between individual NPs, which operate in opposition to the repulsing electrostatic forces.

This observation also highlights the importance of an adequate membrane surrounding the magnetic cores for the colloidal stability of NP suspensions. SAXS profiles of the c-NPs in an aqueous medium are well described with a fractal model of spherical particles with a diameter of 8 ± 2 nm, a fractal dimension of *D_m_* = 2.6 and a cut-off length of 7 nm (Figure S2). The SAXS-derived iron oxide core sizes agree with the cluster sizes derived by semi-quantitative TEM image analysis (Figure 1b).

Phospholipid-coated commercial nanoparticles (p-NPs) also show pronounced aggregation in TEM images (Figure 1c). The supplier datasheet (micromod Partikeltechnologie GmbH) describes the particles as cluster type with a size of 70 nm. TEM images confirm these specifications as they reveal a core diameter in the range of 10 nm and a mean cluster size of 50–100 nm. More detailed analysis of the TEM images does not appear to be purposeful due to the strong tendency towards cluster formation. In view of the functional properties, the supplier provides values of 60 A m^2^ kg^−1^ (iron; *H* = 80 kA/m) and >96 A m^2^ kg^−1^ (iron; *H* > 800 kA/m) for the magnetization and saturation magnetization. SAXS analysis on the phospholipid-coated samples shows, analogous to the SAXS data for c-NPs, clustered particles with core diameters in the range of ~8 nm (Figure S2). Again, this agrees with the magnetite core sizes determined from the TEM images. Based on DLS measurements, the clusters have a mean size of 74.0 ± 1.6 nm and *ζ* potential measurements yield a value of −52.4 ± 1.9 mV (Figure 1d). The conspicuous similarity of these values to those for the b-NPs is expected, as the membrane of magnetosomes from *M. gryphiswaldense* also consist of phospholipids (accompanied by a high protein portion). Accordingly, the swelling behavior of the biogenic magnetite NPs and the commercial p-NPs in water is comparable. It is, however, interesting to note that b-NPs still show a higher colloidal particle stability, i.e., no obvious tendency towards the aggregation of single NP units was observed in TEM images, even though their shell is chemically more complex than the simple phospholipid layer of a p-NP.

### 2.2. Stability Comparison of Commercial and Biogenic Iron Oxide Nanoparticles

In view of biomedical applications, an important aim of stability studies is to analyze the tendency for iron oxide nanoparticle aggregation in cell culture media, which are characterized by an overall complex composition. The commonly used “Roswell Park Memorial Institute” (RPMI) medium and “Dulbecco’s Modified Eagle Medium” (DMEM) were selected as representative cell culture media. Typical NP concentrations used in biomedical applications and scientific studies addressing the cellular level are very low (in the range of mg Fe kg^−1^ body weight). At the same time, the colloidal stability and biocompatibility must be ensured in the accompanying quality control measurements required for each individual particle charge. Accordingly, large-scale instruments such as TEM and SAXS, as well as time-consuming methods such as particle sedimentation assays, are not suited for routine measurements in a clinical environment. Therefore, the use of widely available standard analytic tools, such as those routinely implemented in commercial DLS instruments, may be an ideal alternative for the on-site assessment of colloidal NP dispersions. To categorize the stability and the aggregation behavior of iron oxide NPs in aqueous media by using DLS, we classify particles as “stable”, “metastable” or “unstable in suspension” depending on their determined average *Z*-value (for calculations, please refer to the Supporting Information and Table S2).

Specifically, a particle suspension is defined as stable for *Z* < 2*d.* It is assumed that below this value no aggregation takes place, as four spheres with a diameter of *d* forming a closely packed structure will result in an aggregate with a maximum extension of 2*d* in one dimension. We further define a metastable regime with 2*d* ≤ *Z* ≤ 3*d* because the analyzed particles are not perfectly spherical and, additionally, polydisperse. Accordingly, particles will be referred to as “unstable in suspension” if *Z* > 3*d*. Exemplary DLS correlograms of a stable, a metastable and an unstable NP suspension are given in the SI (Figure S3).

#### 2.2.1. Stability in Culture Media

**Figure 2:**
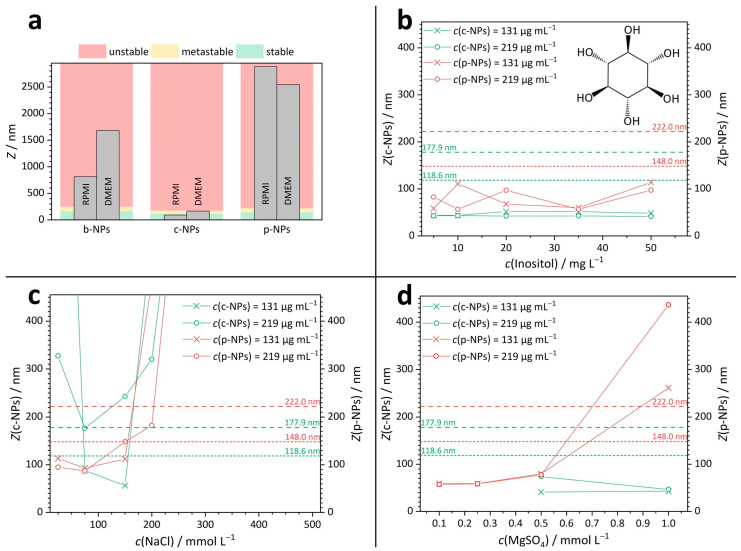
DLS results for iron oxide nanoparticle suspensions. (**a**) Stability of b-NP, c-NP and p-NP suspensions in RPMI and DMEM. (**b**–**d**) *Z*-mean values for c-NPs and p-NPs in aqueous solutions of inositol ((**b**), structure as inset), NaCl ((**c**), for full graph see Figure S4) and MgSO_4_ (**d**) with various concentrations. The dashed lines show the limits for the metastable (short-dashed line) and unstable regions (long-dashed line).

The DLS results for the different iron oxide nanoparticles in the culture media RPMI and DMEM are summarized in Figure 2a; the corresponding *Z*-values are provided in the Supporting Information (Table S3). The respective NP concentrations in the cell culture media ranged from 62 µg mL^−1^ to 131 µg mL^−1^. The color code refers to the different stability regimes. In detail: green indicates stable, yellow indicates metastable and red indicates unstable dispersions. DLS measurements showed that stable suspensions were only formed by c-NPs with a *Z*-mean of 91 nm in RPMI (Figure 2a). In contrast, both p-NP suspensions (*Z* = 2884 nm) and b-NP suspensions (*Z* = 812 nm) were unstable in RPMI. Similar results were obtained for DMEM, where c-NP suspensions with a *Z*-mean of 165 nm were found to be metastable and p-NP (*Z* = 2543 nm) and b-NP suspensions (*Z* = 1684 nm) were again classified as unstable.

To estimate the influence of the individual components of the cell culture media, DLS measurements were performed on simplified solutions (Figure 2b–d), such that the complexity of the media was reduced to a single ion or molecular species—exemplary salt concentrations present within the culture media amount to *c*(NaCl) = 110 mmol L^–1^ in RPMI and *c*(NaCl) = 103 mmol L^–1^ in DMEM and *c*(MgSO_4_) concentration = 0.81 mmol L^–1^ in RPMI and *c*(MgSO_4_) = 0.41 mmol L^–1^ in DMEM. More comprehensive information on the cell culture media compositions and the DLS-derived values for the sizes of NPs and/or aggregates in inositol, NaCl and MgSO_4_ solutions are given in the SI (Tables S4 and S5).

***p-NPs and c-NPs in inositol solutions***

Vitamins and sugars play an important role as small molecular components in cell culture media. Inositol was selected as a representative of this substance class, and the stability of NP suspensions in aqueous inositol solutions was tested (Figure 2b). For inositol concentrations in the range between 5 and 50 mg L^–1^, all measured *Z*-means for the analyzed suspensions were found to be in the stable regime. Hence, we propose that the instability of NP suspensions in complex cell culture media was presumably not caused by their charge-neutral organic components but more likely can be traced back to the presence of ionic species whose influence is discussed below.

***p-NPs in salt solutions***

In aqueous sodium chloride (NaCl) solutions with a salt concentration in the range between 25 and 500 mmol L^−1^ (Figure 2c), suspensions of p-NPs were stable only up to a NaCl concentration of around 200 mmol L^−1^ (red curves in Figure 2c). Note that the NaCl concentration in the cell culture media is lower than this value. In detail, for a p-NP suspension with an iron species (Fe) concentration of 219 µg mL^−1^, metastability was observed at a NaCl concentration of 200 mmol L^−1^ and instability occurred at 300 mmol L^−1^ and above. Suspensions of p-NPs with an Fe concentration of 131 µg mL^–1^ skipped the metastable range entirely and became unstable at NaCl concentrations above 200 mmol L^−1^.

A higher ionic strength in the surrounding media leads to a higher compression of the electrostatic double layer (on the NP surface). Therefore, the *Z*-mean values in the stable concentration regime determined in monovalent salt (NaCl) solution should be significantly larger than those observable in divalent salt solutions (e.g., MgSO_4_). Indeed, p-NPs in aqueous MgSO_4_ (red curves in Figure 2d) had already exceeded the stable and the metastable region in a MgSO_4_ concentration range between 0.5 and 1.0 mmol L^−1^. The phospholipid-coated p-NPs further showed a steady increase in *Z*-mean values in salt solutions with increasing MgSO_4_ concentrations. This relationship was true for both Fe concentrations: 131 µg mL^–1^ and 219 µg mL^–1^. Thus, the contribution of (multivalent) ions in the culture and application media is indeed a crucial factor to be considered; if possible, the number of counter ions in the surrounding medium should be reduced to ensure colloidal stability.

***c-NPs in salt solutions***

The citric-acid-coated c-NPs are slightly smaller than p-NPs. Interestingly, there were no conditions identified under which c-NP suspensions with an Fe concentration of 219 µg mL^–1^ were stable (green curves in Figure 2c). For c-NPs with an Fe concentration of 131 µg mL^–1^, there was a stable “window” for NaCl concentrations between 75 and 150 mmol L^–1^. At NaCl concentrations below 75 mmol L^–1^, no stable c-NP suspension exists. This observation indicates that even the NP concentration can shift the *Z*-mean, as NP–NP interactions restrict the electrophoretic mobility (mm^2^ s^−1^ V^−1^) and thus also determine the maximum amount of NP content available in applications. In aqueous magnesium sulfate (MgSO_4_) solutions with a concentration between 0.1 and 1.0 mmol L^–1^ (Figure 2d), suspensions of c-NPs (green curves in Figure 2a) with Fe concentrations of 131 µg mL^–1^ or 219 µg mL^–1^, respectively, were stable. This is in contrast to the results for p-NPs and can be explained by the significantly higher surface charge of c-NPs (*ζ*_c-NP_ = −111 mV, *ζ*_p-NP_ = −52 mV).

In summary, DLS and *ζ* potential measurements have proven to be valuable tools for the estimation of NP stability in selected media under given specifications. Please note that the *Z*-value is an intensity-weighted mean of the hydrodynamic diameter of a sample, in which larger sizes are weighted significantly more strongly and thus, the contribution of large particles is emphasized. A closer look at the DLS correlograms (Figure S5) shows that in some suspensions, a number of (larger) aggregates exist that are in the size range >1000 nm, where the NPs would become too large to reach the target regions in biomedical/clinical applications [4]. Consequently, the number of available and active NPs would be significantly reduced under these conditions. Hence, the results presented here must be interpreted with caution. However, if the *Z*-mean is still in the stable region despite these constraints, the probability of potential complications due to non-ideal-sized NPs is expected to be low. Nevertheless, for quality control assessment it may be useful to routinely specify the particle size fractions and their individual proportion in addition to the *Z*-mean.

#### 2.2.2. Influence of the Biological Shell

In biomedical applications, NPs are exposed to complex physiological fluids, with a large variety of diverse sugars, vitamins and proteins at different concentrations [38]. An (non-specific) attachment of biological macromolecules to the NP’s surface, i.e., the formation of a (protein) corona, is the consequence [39,40]. Such NP–protein complexes greatly differ from the “naked” NPs with regard to their physicochemical features, such as the surface charge, overall size and functionality [41]. Hence, the stability of NPs in physiological fluids/cell culture media strongly depends on the generated protein corona [42]. In many envisioned biomedical/clinical NP-based applications, the NPs directly enter the bloodstream, in which the protein human serum albumin (HSA) is the most abundant protein [43]. HSA is a monomeric, multidomain macromolecule that is mainly responsible for modulating the fluid distribution between body compartments [43]. Its strong capacity towards ligand binding makes it one of the most important carrier molecules [44]. Adsorbed protein extensions on the particle surface are responsible for the interstitial hydration phenomena between the NPs and the fluid phase and are therefore critical for body fluid balance. In a protein corona the charge distribution may vary, for example by a (partial) neutralization of negatively charged residues of HSA at physiological pH, which may result in a significantly swollen biological shell. Thus, the pH and the surface charge are crucial influences on the stability.

The pronounced ligand binding properties suggest that HSA may be one main component of the protein corona surrounding NPs when entering the human bloodstream. The entropy gain related to the counterion release upon the binding of the protein is thereby a driving force. It is highly likely that the bound molecules, which constitute the corona, will mediate the subsequent (cell) interaction in potential applications.

Bacterial b-NPs are assumed to exhibit a preassembled protein corona acquired during magnetosome isolation from disrupted cells of *M. gryphiswaldense*. Accordingly, in a previous study [19], a variety of non-native magnetosome proteins was identified in the magnetosome membrane fraction of purified particles by mass spectrometry. Examples include cytoplasmic proteins as well as proteins known to be exclusively localized in the bacterial cell wall. In TEM micrographs (Figure S1), the magnetite cores are surrounded by an electron-light organic layer of 3–5 nm in thickness that represents the magnetosome membrane. In negatively stained magnetosome preparations, however, a significantly thicker electron-light layer of up to 15 nm became visible, which suggests the formation of a biological shell (which is not visible in unstained preparations; Figure S1). Nevertheless, the extent to which the hypothesized preassembled protein corona on the b-NP surface interacts with cell culture media or physiological fluids still has to be investigated.

The formation of a protein corona in cell culture medium on the surface of artificial NPs has been described in many studies. For instance, Gräfe et al., (2016) incubated polyethyleneimine-coated magnetic NPs in cell culture media with increasing fetal bovine serum (FBS) concentrations [45]. The results showed that the amount of protein adsorbed to the particle surface clearly increased with the FBS concentration. Similarly, Glancy and co-workers incubated gold NPs of different sizes with human serum and investigated and characterized protein corona formation [46].

The adsorption of molecules on chemically synthesized NPs as well as b-NPs (including their preassembled protein corona) is not limited to serum albumin. However, in a first attempt, p-NPs incubated with HSA can serve as a simple model system to characterize protein adsorption on b-NPs. In addition, c-NPs were used as further model system to investigate how the NP’s surface properties (i.e., type of coating) influence or even trigger/promote protein adsorption and thus corona formation.

***TEM Studies***

**Figure 3:**
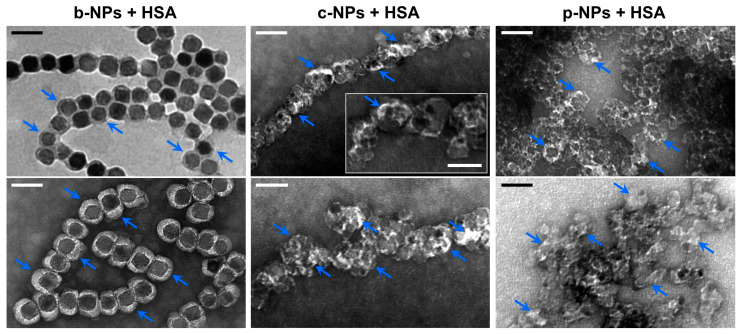
TEM micrographs of different HSA-incubated magnetic NPs. Suspensions of b-NPs (**left**), c-NPs (**middle**) or p-NPs (**right**) (each 826 µg Fe mL^−1^) were incubated with HSA at a final concentration of 10 mg mL^−1^. After removal of excess HSA by performing several washing steps, the particles were analyzed by TEM. For b-NPs, a clear electron-light organic shell had already become visible in the unstained state (**top left**, indicated by arrows), suggesting the presence of a protein corona. In negatively stained preparations (**bottom left**), an even more pronounced well-defined shell could be visualized. In negatively stained samples of c-NPs and p-NPs (each incubated with HSA), an electron-light organic shell became visible as well (blue arrows); however, due to the rather irregular shape of the NPs and their strong tendency to form clusters, the layer thickness cannot easily be assessed in this case. (Scale bar: 100 nm, scale bar inset “c-NP + HSA”: 50 nm).

In order to investigate HSA adsorption on the magnetosome surface, a b-NP suspension (826 µg mL^−1^) was incubated with HSA (10 mg mL^−1^) overnight. TEM micrographs of negatively stained preparations indicated the presence of a partially asymmetric electron-light organic shell of 23.0 ± 5.0 nm in thickness (largest extension 36.3 nm) that surrounds the crystalline magnetite cores. In contrast, for negatively stained preparations without HSA, a layer of only 10.7 ± 4.1 nm could be visualized (Figure S1), suggesting the adsorption of HSA molecules onto the preassembled magnetosome protein corona in the former case. Remarkably, even for unstained preparations an organic layer of clearly increased thickness (compared with the “pristine” magnetosome membrane) became visible (Figure 3, top left, blue arrows), which might indicate the presence of an (hard) HSA corona.

In TEM preparations of negatively stained c-NPs and p-NPs (826 µg mL^−1^), each incubated with HSA at a concentration of 10 mg mL^−1^ overnight, an electron-light shell became visible as well. However, due to the presence of clusters consisting of irregularly shaped particles, the overall thickness of the HSA corona is difficult to assess. Assuming that the bright contrast regions in the TEM micrographs can be mainly attributed to electron-light adsorbed proteins, a shell thickness of up to 12 nm was measured, which is significantly smaller than the value determined for b-NPs (23 nm on average). Therefore, it can be proposed that the preassembled protein corona on the b-NP surface, with its variety of protein and peptides, facilitates the further adsorption of HSA. This is in contrast to the artificial c-NPs and p-NPs, for which such a matrix promoting protein adsorption has first to be formed. Accordingly, the negatively charged citric acid groups on the c-NP surfaces and the phospholipid (lecithin) layer on the p-NP surfaces are less prone to HSA adsorption and the formation of a protein corona. Furthermore, the varying sizes of the NPs may lead to different HSA surface packing densities.

***DLS Studies***

To further investigate the formation of a protein corona, HSA at different concentrations (i.e., 1–10 mg mL^−1^) was added to p-NP or c-NP suspensions in ddH_2_O or HEPES (buffering capacity between pH 6.8 and 8.2). As described above, the *Z*-mean from DLS measurements was used to determine the stability of the NP suspensions. The commercial c-NPs (131–826 μg mL^−1^) were found to be stable in suspension by addition of HSA, whereas p-NPs were stable in HEPES and less stable in water upon addition of the protein (see Figure S5).

**Figure 4:**
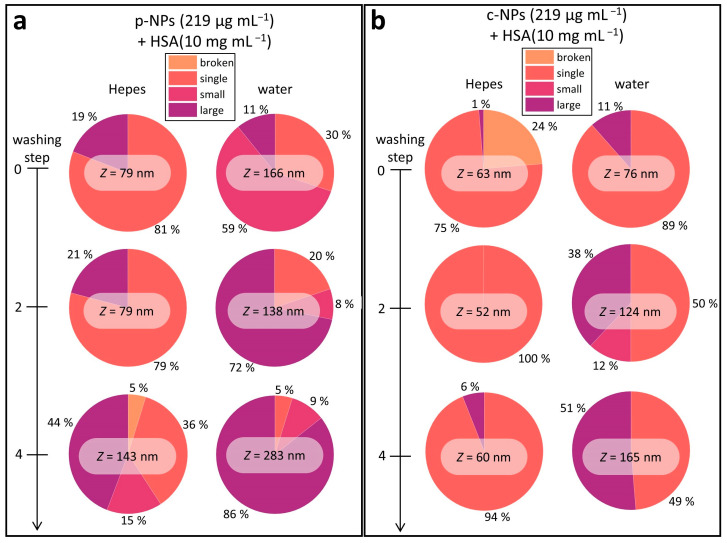
Overview of the *Z*-means and the different size classes in the investigated iron oxide NP suspensions (in HEPES or water). Overall, the NP stability in HEPES is higher than in water, and the number of larger species increases with each washing step. The data on p-NPs (**a**) and c-NPs (**b**) showed a similar trend; however, the latter are more stable compared with p-NP suspensions.

To elucidate the stability of the protein corona, HSA (10 mg mL^–1^) was added to c-NP or p-NP suspensions (219 μg mL^−1^), and the resulting mixtures were then subjected to several washing steps. One washing step consisted of centrifugation, decantation of the supernatant and resuspension of the sediment in the respective solvent (HEPES or water). DLS measurements were performed before washing and after two and four washing steps (Figure 4). In DLS data evaluation, the Z-value, which represents a cumulants mean, is usually considered (as described above). However, for multimodal size distributions this value lacks information about the amount and size of individual particle size classes. A closer look at the measured correlograms often revealed a non-ideal behavior and therefore different size classes (Figure S5). In order to estimate the amount and individual size of the particles and clusters, we categorized object classes as disrupted particle fragments (broken), individual NPs (single), small clusters (small) and large aggregates (large) (Table 1). Figure 4 shows the relative proportion of the different size classes after various washing steps and, additionally, the determined *Z*-value, emphasizing the distorted picture which is given by the *Z*-value regarding the suspension stability. As can be seen, some suspensions (e.g., p-NPs in HEPES in Figure 4a and c-NPs in HEPES in Figure 4b) can be considered as stable by just taking the *Z*-value into account, even if they contain a substantial number of aggregates that are in a critical size range for biomedical or clinical applications. The overall trend of increasing *Z*-values with further washing steps confirms the occurrence of larger aggregates, which shift the size weighted *Z*-mean to higher values. Additional (diagnostic or clinical) studies are required to investigate the number of aggregates that can be tolerated in applications.

Commercial p-NPs were stable in HEPES upon addition of HSA (10 mg mL^–1^), as seen in the DLS results (Figure S6a,b). However, the proportion of large aggregates was 19% (Figure 4a). In water, the fraction of large aggregates was smaller, but there was a huge fraction of small clusters (about 60%). In agreement with the evolution of the *Z*-mean in both cases, the proportion of individual particles decreased continuously with each washing step. In detail, the number of individual p-NPs dropped from 81% to 36% in HEPES and from 30% to 5% in water. In addition to this, after the fourth washing step broken particles occurred in HEPES suspensions.

Commercial c-NP suspensions were also stable in HEPES and water upon addition of HSA (10 mg mL^−1^), as shown by the evaluation of the *Z*-means from DLS measurements (Figure S6c,d). Close examination of the individual sizes revealed few large aggregates of c-NPs in HEPES, with a fraction of only about 6% after four washing steps, i.e., 94% of the individual particles still remained (Figure 4b). c-NPs showed the best stability in water directly after HSA addition. Specifically, individual NPs made up 89% and 11% of the total was in the form of large aggregates. After washing, their stability significantly dropped, reaching 49% for individual NPs and 51% for large aggregates after the fourth washing step.

Overall, the DLS results show an increased stability for the NPs in HEPES buffer and after addition of HSA, which is presumably caused by the formation of a protein corona surrounding the NPs. Further, the HSA corona seems to be more stable for c-NPs compared with p-NPs. However, the stability decreases during washing, possibly due to (partial) depletion of the protein shell.

**Figure 5:**
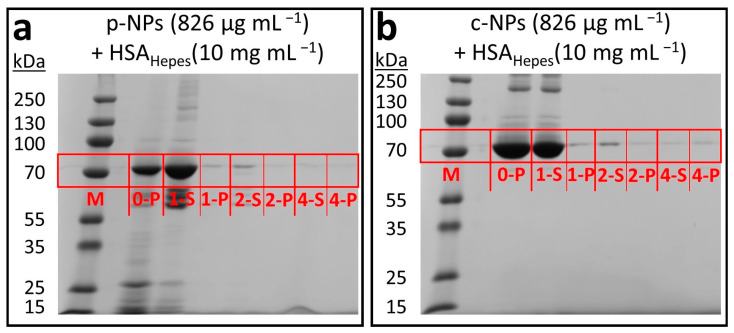
SDS-PAGE results for iron oxide nanoparticle suspensions in HEPES after addition of HSA (10 mg mL^–1^). “M” denotes the lane of the protein molecular weight marker (“prestained plus”); the washing steps are indicated by their respective numbers. “S” indicates the supernatants and “P” the pellet of each sample. (**a**) The protein corona surrounding p-NPs was mostly rinsed off after the first washing step. Consequently, more HSA was found in the supernatant (1-S). The presence of a small band in the pellets even after several washing steps hints to the existence of a hard HSA protein corona (1-P, 2-P and 4-P). (**b**) Similar results were obtained for c-NPs.

Denaturing polyacrylamide gel electrophoresis (SDS-PAGE; Figure 5) was performed to obtain information on the integrity of the HSA protein corona on the particle surface and might be an additional tool to address the stability of the NP suspensions. The composition of the gels and buffers used for SDS-PAGE are given in the Supporting Information (Table S6). HSA-incubated c-NP and p-NP samples were subjected to electrophoresis to monitor the depletion of the generated HSA corona upon several washing steps.

Due to the lack of further proteins in the respective NP suspensions, only HSA was visualized on the SDS gels. The comparison with the protein molecular weight marker (“prestained plus” control) in lane M allows estimation of the molecular mass of HSA, which is about 70 kDa (calculated molecular mass according to amino acid composition 66.5 kDa). Further bands with increased or reduced electrophoretic mobility can be ascribed to not fully denatured HSA oligomers or degradation products potentially generated in the course of sample preparation. A HEPES solution enriched with p-NPs (826 µg mL^–1^) and HSA (10 mg mL^–1^) showed a large amount of HSA before purification (0-P in Figure 5a). After the first washing step, however, most of the proteins were washed off and thus could be found in the supernatant (1-S in Figure 5a), whereas only a small fraction remained in the pellet (1-P in Figure 5a). Further washing steps resulted in a decreasing amount of HSA in the particle fractions (2-P and 4-P in Figure 5a) and a remnant of the protein in the supernatant (2-S and 4-S in Figure 5a). This indicates the presence of an HSA protein corona in both cases that consists mainly of loosely bound HSA (soft corona). Thus, as expected, only a few proteins build-up the so-called hard corona, which consists of strongly adsorbed HSA (seen in fractions 2-P and 4-P in Figure 5a). This ratio is not unexpected, since the space on the NP surface is restricted (spatially and as a consequence of steric hindrances between the attached proteins). Analogous results can be obtained in a HEPES solution enriched with c-NPs (826 µg mL^–1^) and HSA (10 mg mL^–1^) (Figure 5b). Most of the protein corona is rinsed off in the first washing step. These observations are consistent with the decreasing stability of the NP suspensions (cf. Figure 4).

Overall, our physicochemical analyses (in particular TEM and DLS) show that even at low ionic strength and despite its negative net charge, the HSA protein (isoelectric point of 4.7) adsorbed onto the NP surfaces. Hence, positive patches of HSA must become multivalent and strongly bind counterions on the NP surfaces, thereby forming a hard corona and releasing a concomitant number of co- and counterions into the surrounding medium. The repulsive (charge) interaction is thereby counterbalanced by the entropy gain of the entire system. On the hard corona, HSA–HSA interactions may lead to the formation of an additional protein layer, the soft corona. This soft shell can easily be washed off since HSA is only loosely adsorbed. In pure water (low ionic strength), the protein corona, as a biological shell that consists of a hard and a soft protein layer, is expected to be highly “swollen”, since the osmotic pressure of the counterions may lead to a significant unfolding/extension of the protein corona into the solvent. In HEPES (high ionic strength), the electrostatic interaction will be strongly screened and the NP size will be most probably determined by the mutual interaction of the NP surface charges (protein in the folded/more compact state). Thus, the extension of the protein shell will be significantly lower than in water.

## 3. Materials and Methods

### 3.1. Chemicals and Materials

Commercial iron oxide NPs encapsulated by a phospholipid membrane for stabilization (p-NPs) were purchased from micromod Partikeltechnologie GmbH (product code: 45-111-701; Rostock, Germany). Commercial iron oxide NPs coated with a citric acid matrix for stabilization (c-NPs) were purchased from chemicell GmbH (product code: 4122-5; Berlin, Germany). Specifications of the different NP types are displayed in Table S7 in the Supporting Information (SI).

Cell culture media were also commercially purchased: DMEM from HiMedia Laboratories Pvt. Ltd. (Mumbai, Maharashtra, India) and RPMI from Thermo Fischer Scientific (Waltham, MA, USA). Detailed information on the media composition can be found in the SI (Table S4).

Further chemicals were purchased from Carl Roth GmbH (Karlsruhe, Germany) or Merck GmbH (Darmstadt, Germany), with purity grades of at least 98%.

### 3.2. Cultivation of M. gryphiswaldense

For magnetosome (b-NP) production, *M. gryphiswaldense* was routinely grown in modified flask standard medium (FSM; 10 mM HEPES, 15 mM K-lactate, 4 mM NaNO_3_, 0.74 mM KH_2_PO_4_, 0.6 mM MgSO_4_∙7 H_2_O, 50 µM Fe^3+^-citrate, 3 g L^−1^ soy peptone and 0.1 g L^−1^ yeast extract, pH 7.0) at 28 °C [47]. Cultivation was performed in 8 L of FSM in 10 L flasks under moderate shaking (120 rpm) as described previously [48]. Under these conditions, magnetosome biosynthesis is induced, resulting in the formation of up to 40 particles per cell and an overall cellular iron content of up to 4% of the cell dry weight. When the cells reached the late exponential growth phase, they were harvested by centrifugation at 9000× *g*, washed with a washing buffer (buffer 1; 20 mM HEPES, 5 mM ethylenediaminetetraacetate (EDTA), pH 7.4) and stored at −20 °C until further use [49].

### 3.3. Magnetosome Purification

Magnetosomes from *M. gryphiswaldense* were isolated as described previously [10,19]. Briefly, approximately 7 mL of buffer 2 (50 mM HEPES, 1 mM EDTA, pH 7.4) was used to resuspend 1 g of cells (wet weight). Three to five grams of cells were then disrupted by 3–5 passages through a microfluidizer system (M-110 L, Microfluidics Corp., Westwood, MA, USA) equipped with an H10Z interaction chamber at 124 MPa. Crude extracts were subsequently passed through a MACS magnetic separation column (5 mL; Miltenyi, Bergisch Gladbach, Germany) that was placed between two neodymium-iron-boron magnets (each 4.0 cm × 2.0 cm × 1.0 cm, 1.3 T) to isolate the particles. Thereby, a field strength of ~400 mT could be achieved within the MACS column. Next, the column was washed with 10 column volumes (CV) of buffer 3 (10 mM HEPES, 1 mM EDTA, pH 7.4), followed by 10 CV of buffer 4 (10 mM HEPES, 1 mM EDTA, 150 mM NaCl, pH 7.4) and again 10 CV of buffer 3 to remove cellular debris and electrostatically bound contaminants. Finally, the magnets were removed from the column to elute the magnetosomes using ddH_2_O. This suspension was further purified by ultracentrifugation through a 60% (*w*/*v*) sucrose cushion (three volumes magnetosome suspension, one volume sucrose) at 200,000× *g* for 2 h at 4 °C. Due to their high density, the magnetosomes pelleted at the bottom of the tube, whereas residual cellular constituents were retained by the sucrose cushion. Finally, the particles were resuspended in buffer 3 and stored in Hungate tubes at 4 °C under a nitrogen atmosphere until further use.

### 3.4. Structural and Compositional Characterization of Nanoparticles

The structure and composition of iron-oxide-nanoparticle-containing suspensions were analyzed using a wide range of imaging, scattering and spectroscopy methods, as well as gel electrophoresis.

For transmission electron microscopy (TEM) analysis of bacteria and isolated NPs, specimens were directly deposited onto carbon-coated copper grids (Science Services, Munich, Germany). In addition, isolated particles were negatively stained with 2% uranyl acetate. TEM was performed on a CEM 902A (Zeiss, Oberkochen, Germany) with an acceleration voltage of 80 kV. Images were recorded with a Gatan Erlangshen ES500W CCD camera.

Nanostructural analyses were performed on highly concentrated particle suspensions under ambient conditions using a Double Ganesha AIR system (SAXSLAB/XENOCS). Monochromatic radiation with a wavelength (*λ*) of 1.54 Å was generated by a rotating anode source (Cu; MicroMax 007HF; Rigaku Corporation, Japan). All data sets were recorded with a position-sensitive detector (Pilatus 300K; Dectris) placed at different distances from the sample to cover a wide range of scattering vectors *q*, with 0.002 Å^−1^ < *q* < 0.5 Å^−1^, where *q* is given as

(1) $$q=\left|\vec{q}\right|=\frac{4\pi }{\lambda }\sin\left(\frac{\theta }{2}\right)$$

where *λ* represents the wavelength of the incident beam and *θ* the scattering angle. The two-dimensional scattering patterns were converted into one-dimensional intensity profiles of *I*(*q*) versus *q* by radial averaging and subsequently normalized to the intensity of the incident beam, the sample thickness and the accumulation time. Background correction was performed by subtracting the signal of a solvent-filled glass capillary (Ø = 1 mm; Hilgenberg, Germany). The software SasView 4.2.2 was used for data analysis.

The iron contents of NP suspensions were determined by atomic absorption spectroscopy (AAS). For these measurements, 10–25 μL of the corresponding suspensions were mixed with 69% nitric acid (final volume 1.0 mL) and digested at 98 °C for 3 h. Sample volumes were adjusted to 3 mL with ddH_2_O and subsequently analyzed using a contrAA300 high-resolution atomic absorption spectrometer (Analytik Jena, Jena, Germany) equipped with a 300 W xenon short-arc lamp (XBO 301, GLE, Berlin, Germany) as the continuum radiation source. The equipment presented a compact high-resolution double monochromator (consisting of a prism pre-monochromator and an echelle grating monochromator) and a charge-coupled device (CCD) array detector with a resolution of about 2 pm per pixel in the far ultraviolet range. Measurements were carried out at a wavelength of 248.3 nm using an oxidizing air/acetylene flame. The number of pixels of the array detector used for detection was 3 (central pixel 1). Iron contents are given as mean values and represent the averaged values of three experiments measured in quintuplicate (*n*_total_ = 15). Please note that all given concentrations refer to the concentration of the iron species.

Zeta potential values and particle sizes (i.e., hydrodynamic diameters of isolated NPs and particle agglomerates) were determined using a Zetasizer Nano-ZS (Malvern, UK). Measurements were performed in the automatic mode at 25 °C on diluted particle suspensions. Each sample was analyzed in quintuplicate on three biological replicates (*n*_total_ = 15) using DTS1070 cuvettes (Malvern, UK). Data evaluation was performed with the software provided by the supplier (Malvern Zetasizer Software 7.13).

Denaturing polyacrylamide gel electrophoresis (PAGE) was performed according to the method described by Laemmli [50] and modified according to Fling and Gregerson [51]. Gels consisted of a 5% (*w*/*v*) acrylamide stacking gel and an 8% → 22.5% (*v*/*w*) gradient running gel. NP suspensions corresponding to 826 µg mL^−1^ Fe were incubated in 4x Laemmli sample buffer (325 mM Tris/HCl pH 6.8, 40% glycerol, 400 mM dithiothreitol (DTT), 8*%* sodium dodecyl sulfate (SDS)*,* 0.01% bromophenol blue) for 15 min at room temperature in order to solubilize proteins bound to the NP surface, which were subsequently separated by electrophoresis.

## 4. Conclusions

In our study, we assessed the stability of different types of magnetic iron oxide NPs in cell culture media (RPMI and DMEM). In order to evaluate the effects of individual compounds, we furthermore incubated the NPs with solvents supplemented with major media components such as salt or inositol (a carbocyclic sugar/vitamin that is known to be a component of membrane phospholipids and an important second messenger in eukaryotic cells [52]), revealing that the stability is mainly influenced by the ionic strength (salt). Note that the salt concentration in RPMI and DMEM is below the critical values for aggregation and thus, NPs suspended in these standard media can be considered as stable.

Additionally, the stability of NP suspensions depends on the formation of a protein corona that is inherently formed in physiological fluids. Even for the artificial c-NPs and p-NPs, protein corona formation was observed. Although the p-NPs were assumed to resemble the b-NPs to some extent (i.e., iron oxide core and phospholipid envelope), the observed thickness of the protein corona turned out to be significantly lower for the synthetic particles than for the b-NPs, most probably due to the fact that b-NPs had already built a preliminary protein corona during isolation from *M. gryphiswaldense* cells. However, not all proteins interact with or adsorb to the NP surfaces in the same manner, allowing the formation of a soft and a hard (more tightly bound) protein corona. This observation is in agreement with previous studies [40,41,53]. The parameters influencing the formation of a protein corona are manifold and depend not only on the particle size and the NP surface characteristics but also on the composition of the medium used to suspend the iron oxide particles [39,54,55,56].

So far, only a few studies have investigated protein corona formation on magnetosome-based NPs. Upon exposure to plasma fractions, a variety of serum proteins was found to be selectively adsorbed onto the particle surface [57], which is in agreement with the results presented in our study. However, for many applications in the (bio)medical or clinical fields, functionalized NPs might be highly beneficial (e.g., NPs that display specific ligands or fluorophores on their surfaces) and the formation of such a protein corona might shield/cover these moieties, leading to a (partial) loss of functionality [58]. Thus, when considering magnetosomes for applications in a biological context, the impact of the protein corona on the particle “behavior” has to be considered and should be ideally utilized and exploited for optimal application. Further studies addressing the enhanced complexity of the magnetosome membrane and the influence of the adsorbed proteins after exposure to physiological fluids/cell culture media are sine qua non.

## Figures and Tables

**Table 1:** Overview of the defined size classes for c-NPs and p-NPs. The size classes depend on the individual particle sizes and are categorized as given in the table.

	Broken	Single	Small	Large
c-NPs	<20 nm	20–100 nm	100–400 nm	>400 nm
p-NPs	<40 nm	40–140 nm	140–500 nm	>500 nm

## Data Availability

Not applicable.

## Supplementary Materials

The following supporting information can be downloaded at: https://www.mdpi.com/article/10.3390/molecules28134895/s1, Figure S1: Illustration of magnetosome structure and biosynthesis in *M. gryphiswaldense*; Figure S2: SAXS data for commercial nanoparticles; Figure S3: Exemplary DLS correlograms of stable, metastable and unstable samples; Figure S4: *Z*-mean values of c-NPs and p-NPs, respectively, in aqueous NaCl solutions; Figure S5: Exemplary DLS correlograms and size distribution profiles of samples with monomodal and multimodal size distributions; Figure S6: DLS results for commercial iron oxide nanoparticles in HEPES and water with addition of HSA in various concentrations; Table S1: Iron oxide nanoparticle core sizes determined with imaging and scattering methods; Table S2: Comparison of *Z*-means and size values *d* for differently concentrated NPs in water; Table S3: DLS-derived particle sizes of iron oxide NPs in RPMI and DMEM; Table S4: Detailed composition of the cell culture media used; Table S5: Particle sizes of iron oxide NP suspensions in different media as determined by DLS; Table S6: Composition of the gels and buffers used for SDS-PAGE; Table S7: Product information of commercial iron oxide nanoparticles as specified by the manufacturers. References [59,60,61,62,63,64] can be found in the Supplementary Materials.

## Sample Availability

Samples of the compounds are not available from the authors.
