# Supplementary material for: Comparing the Colloidal Stabilities of Commercial and Biogenic Iron Oxide Nanoparticles That Have Potential In Vitro/In Vivo Applications
Source: Molecules. 2023 Jun 21;28(13):4895. doi: 10.3390/molecules28134895 (PMC10343720; doi:10.3390/molecules28134895)
Supplement: Supplementary file 1 [file molecules-28-04895-s001.zip › molecules-2407293-supplementary.pdf]

Comparing the Colloidal Stabilities of Commercial and Biogenic Iron Oxide Nanoparticles that have Potential In Vitro / In Vivo Applications

Jonas Schwan[1,†], Simon Markert[2,†], Sabine Rosenfeldt[3,4], Dirk Schüler[2], Frank Mickoleit[2,*] and Anna S. Schenk[1,4,*]

[1] *Physical Chemistry IV, University of Bayreuth, D-95447 Bayreuth, Germany*
[2] *Dept. Microbiology, University of Bayreuth, D-95447 Bayreuth, Germany*
[3] *Physical Chemistry I, University of Bayreuth, D-95447 Bayreuth, Germany*
[4] *Bavarian Polymer Institute (BPI), University of Bayreuth, D-95447 Bayreuth, Germany*

† *These authors contributed equally to this work.*
* *Correspondence: frank.mickoleit@uni-bayreuth.de (F.M.); anna.schenk@uni-bayreuth.de (A.S.S.)*

Supplementary Data and Analyses

Analyses

Details on the calculation of the *Z*-mean value

Figures

Figure S1 Illustration of magnetosome structure and biosynthesis in *M. gryphiswaldense*
Figure S2 SAXS data of commercial nanoparticles
Figure S3 Exemplary DLS correlograms of stable, metastable, and unstable samples
Figure S4 *Z*-mean values of c-NPs and p-NPs, respectively, in aqueous NaCl solutions
Figure S5 Exemplary DLS correlograms of samples with monomodal and multimodal size distributions
Figure S6 DLS results for commercial iron oxide nanoparticles with addition of HSA

Tables

Table S1 Iron oxide nanoparticle core sizes determined with imaging and scattering methods
Table S2 Comparison of *Z*-means and size-values *d* for differently concentrated NPs in water
Table S3 DLS-derived particle sizes of iron oxide NPs in RPMI and DMEM, respectively
Table S4 Detailed composition of the used cell culture media
Table S5 Particle sizes of iron oxide NP suspensions in different media as determined by DLS
Table S6 Composition of the gels and buffers used for SDS-PAGE
Table S7 Product information of commercial iron oxide nanoparticles as specified by the manufacturers

Analyses

Details on the calculation of the Z-mean value

Dynamic light scattering (DLS) is a well-established measurement technique for the characterization of particle sizes in suspension and is based on the Brownian motion of the particles within the medium. Temporal intensity fluctuations of the scattered light depend on the velocity of diffusion, which can be related to the hydrodynamic size distribution by mathematical descriptions. As a stable parameter, which can be extracted from DLS measurements, the *Z*-average is often used for quality assessments, since it is sensitive to small changes in the size distribution of the sample, for example due to the formation of a small number of aggregates or clusters. The *Z*-mean is only strictly associated to an averaged particle size *d* in case of homogeneous spherical particles showing only one narrow peak in the size distribution (monomodal) or for polydisperse samples in which the main peak/main contribution in the size distribution fulfills that criterion. In Table S2 both common parameters, *d* and *Z*-value, are exemplarily compared.

The *Z*-mean represents the intensity-weighted average hydrodynamic size of the measured particles. The calculation is described below, where Z represents the *Z*-mean, S_i represents the intensity scattered by particle i, and D_i represents the diameter of particle i:

$$Z = \frac{\sum S_i}{\sum \frac{S_i}{D_i}}.$$

For the case of sufficiently small particles (Rayleigh scatterers), the relationship $S_i \approx {D_i}^6$ is valid. Therefore, the *Z*-mean can be approximated as follows:

$$Z \approx \frac{\sum {D_i}^6}{\sum {D_i}^5}.$$

The *Z*-mean of a polydisperse sample is calculated using the cumulant analysis method, which is based on a numerically stable least squares fit, and thus is relatively insensitive to experimental noise. In the analysis, the autocorrelation function $C(\tau)$ (τ: correlation time) is considered:

$$C(\tau) = A + B\,\exp(-2\bar{\Gamma}\tau),$$

with A and B as fit parameters and

$$\bar{\Gamma} = D_{t,\mathrm{avg}} \times q^2,$$

where $\bar{\Gamma}$ is the mean relaxation rate and $D_{t,\mathrm{avg}}$ represents the intensity-weighted average diffusion coefficient. The scattering vector q is given by the equation:

$$q = \frac{4\pi n}{\lambda} \cdot \sin\left(\frac{\theta}{2}\right).$$

Here, n represents the refractive index, λ is the wavelength of the laser, and θ represents the scattering angle. The Stokes-Einstein equation is then used to infer the average particle size Z from $D_{t,\mathrm{avg}}$:

$$Z = \frac{k_B T}{3\pi\eta D_{t,\mathrm{avg}}},$$

where k_B is the Boltzmann's constant, T the thermodynamic temperature, and η the dynamic viscosity. For a more detailed description of the *Z*-mean calculation from DLS data sets please refer to ISO-22412:2017.[1]

Figures

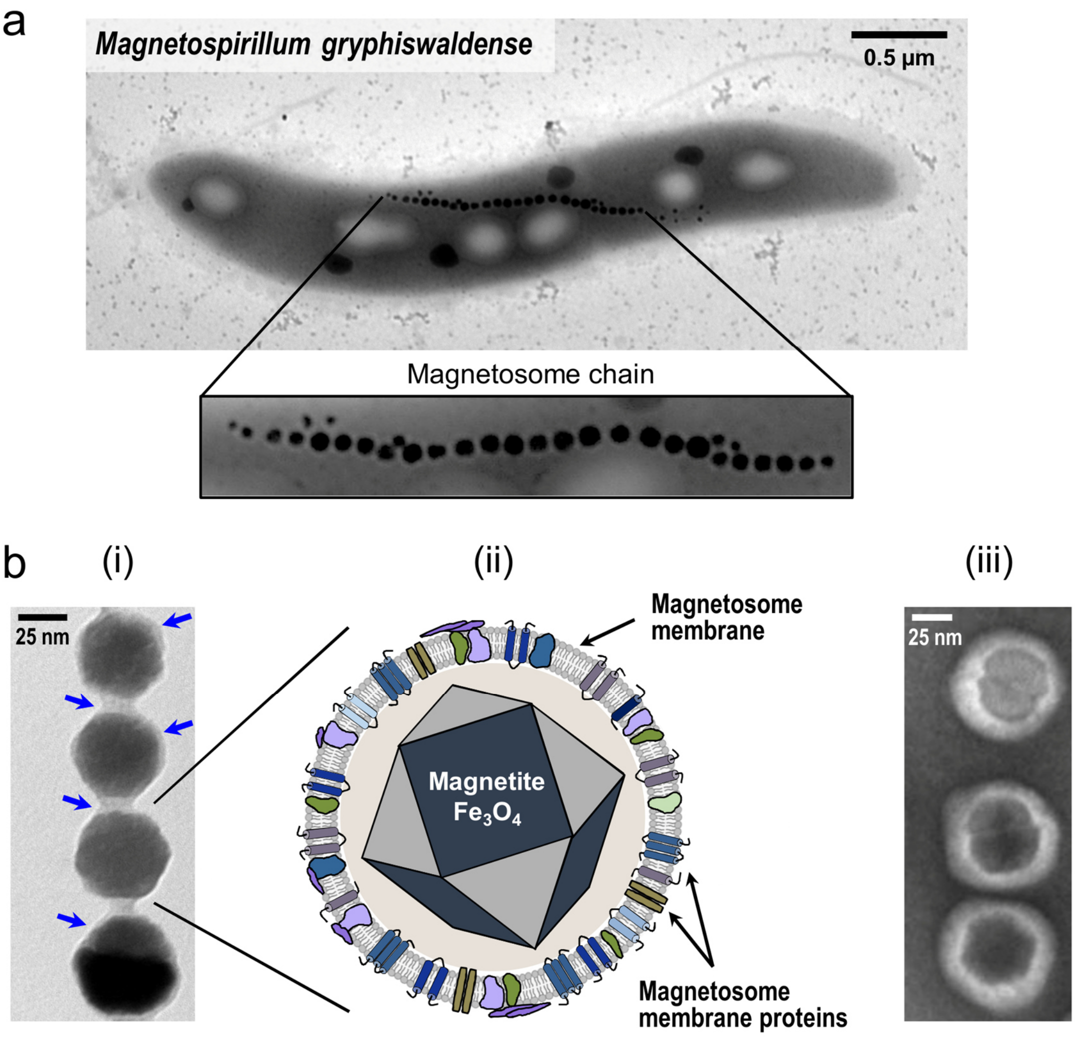

Figure S1 Illustration of magnetosome structure and biosynthesis in *M. gryphiswaldense.* (a) TEM micrograph of a wildtype cell of *M. gryphiswaldense* (scale bar: 0.5 µm). Under microoxic / anoxic cultivation conditions, up to 40 magnetosomes are biomineralized, arranged in a chain-like manner at midcell (magnification). (b) Magnetosome suspensions contain particles with uniform shape and a narrow particle size distribution (i), and can be isolated from disrupted cells, the intact magnetosome membranes are indicated by blue arrows. (ii) Magnetosomes consist of a cuboctahedral core of chemically pure magnetite that is surrounded by a phospholipid bilayer (magnetosome membrane). The latter harbors a set of specific proteins (magnetosome membrane proteins) that fulfill essential functions in magnetosome biosynthesis. (iii) In TEM micrographs of negatively stained preparations, an electron-light organic shell of increased thickness (compared to the magnetosome membrane highlighted in (i)) became visible, suggesting the formation of a pre-assembled protein corona on the magnetosome membrane during particle isolation (for more details please refer to the main text).

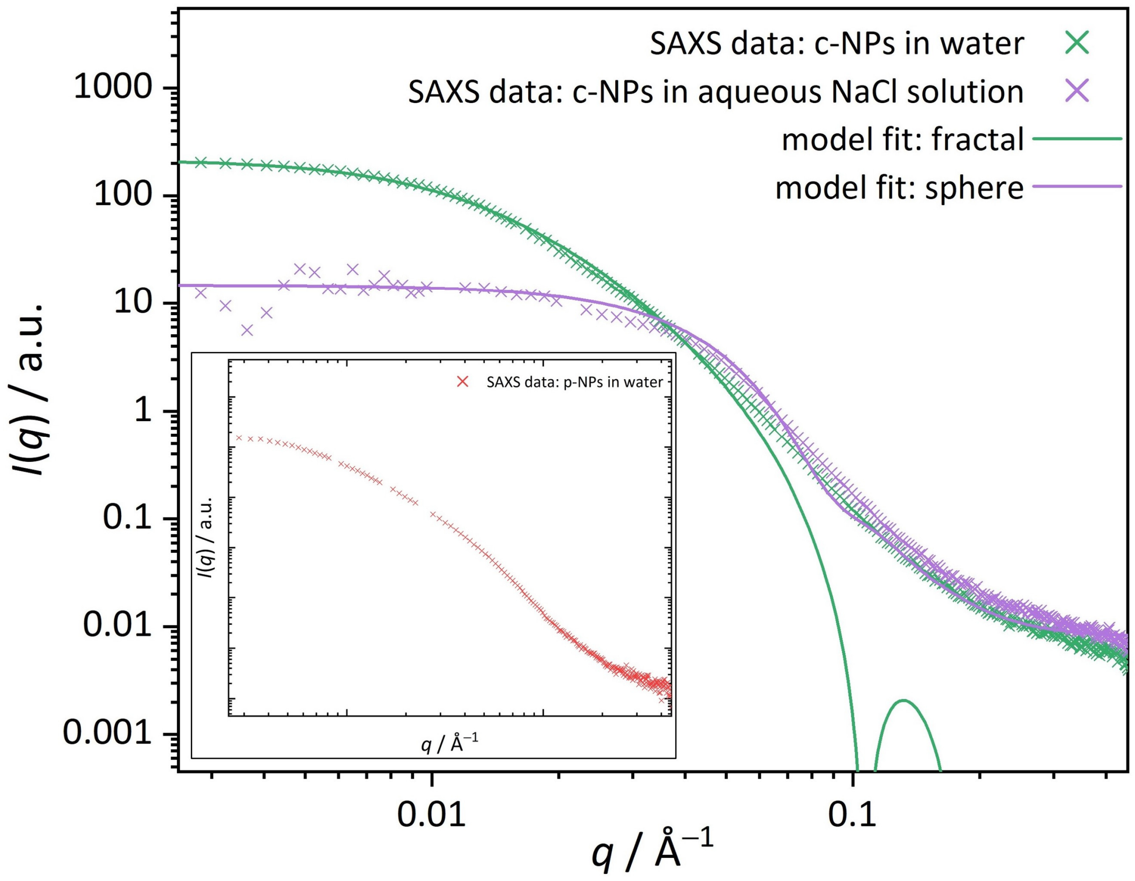

Figure S2 SAXS data of commercial nanoparticles. Experimental data of c-NPs (25 mg mL^{-1}) in water (green crosses) can be described by a mass fractal model[2] (green line) with a particle radius $R = 4.2$ nm, a fractal dimension $D_m = 2.6$ and a cut-off length of 7 nm, the latter being an equivalent to the gyration radius of the fractal. Additionally, SAXS data of c-NPs in an aqueous NaCl solution under conditions for which we only expect individual, non-aggregated particles are shown (purple crosses). Here, a model for hard spheres[3] with a radius $R = 4.2$ nm and a gaussian polydispersity of 25% fits to the raw data. Inset: SAXS data of p-NPs (10 mg mL^{-1}) in water (red crosses) which show strong similarities to the c-NPs.

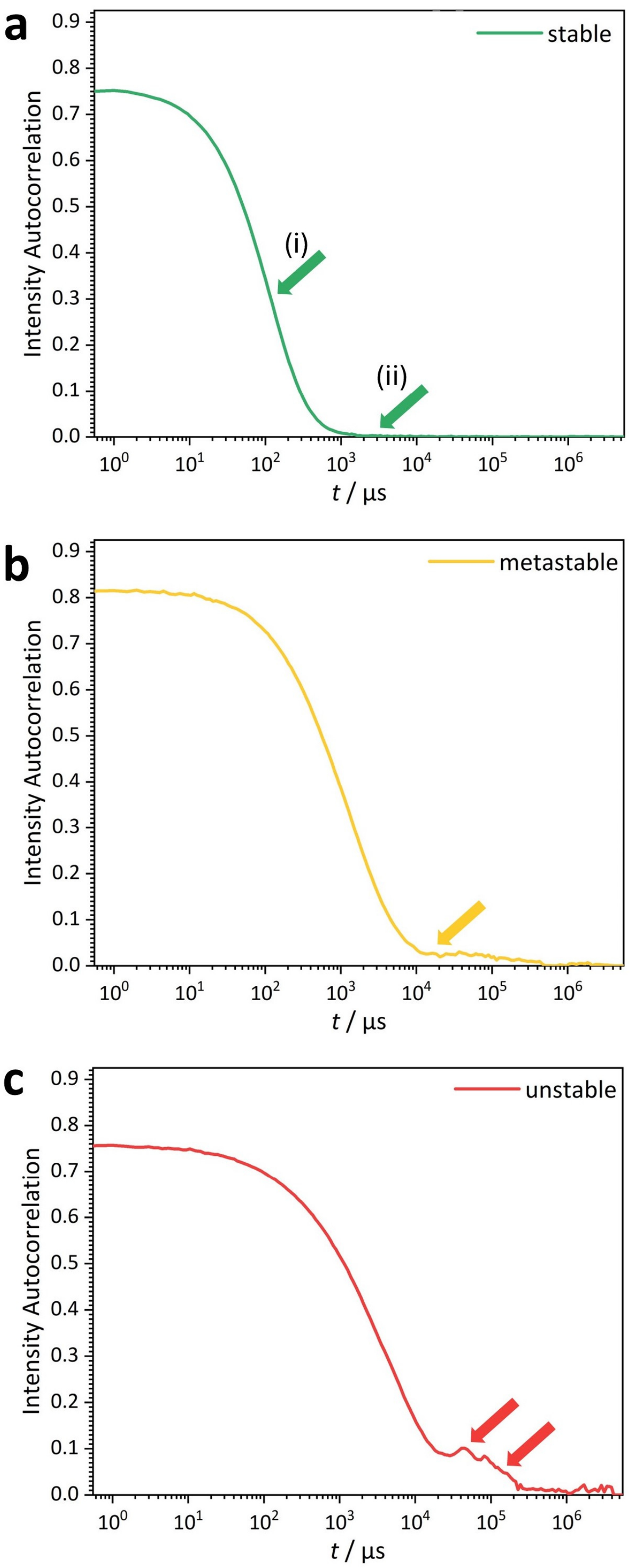

Figure S3 Exemplary DLS correlograms of stable, metastable, and unstable samples, respectively. (a) c-NPs (c(NPs) = 88 µg mL^{-1}) in RPMI (stable), (b) c-NPs (c(NPs) = 62 µg mL^{-1}) in DMEM (metastable), (c) b-NPs (c(NPs) = 88 µg mL^{-1}) in DMEM (unstable). DLS profiles corresponding to stable samples are characterized by a single decay within the correlation time (i) and regression to the baseline (ii) (marked by the green arrows). The yellow arrow indicates the missing regression to the baseline in the data of the metastable sample. The red arrows mark the parts of the correlogram where the requirements of a stable measurement are not met. Here, a second decay is visible and the correlogram lacks a sufficient regression to the baseline.

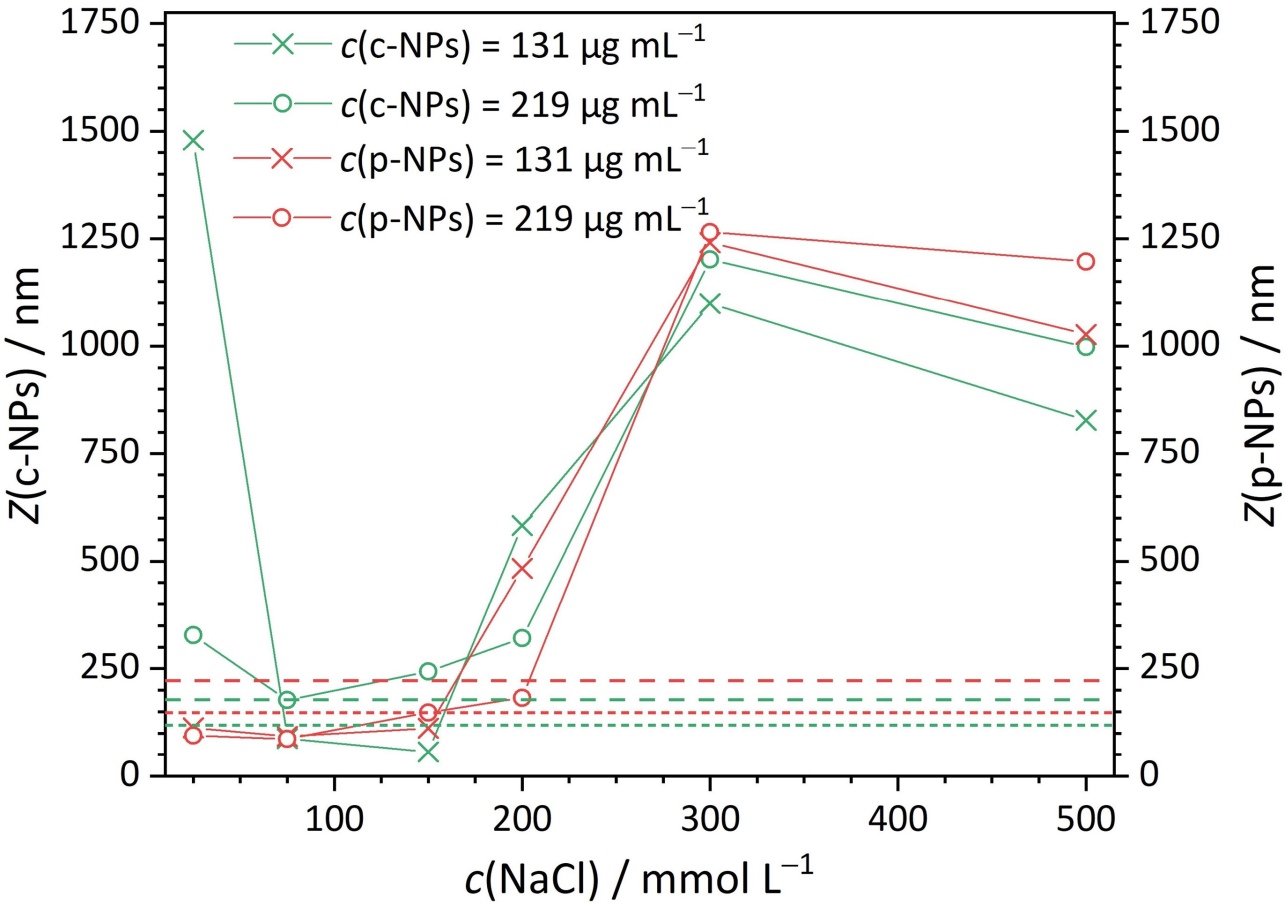

Figure S4 *Z*-mean values of c-NPs and p-NPs, respectively, in aqueous NaCl solutions. The dashed lines indicate the limits for the metastable and unstable regions. For c-NPs stable region extends to a value of 118.6 nm and particles with a *Z*-value above 177.9 nm are labeled as unstable. For p-NPs the equivalent values are 148.0 nm and 222.0 nm, respectively. The dashed lines show the limits for the metastable (short dashed line) and unstable regions (long dashed line).

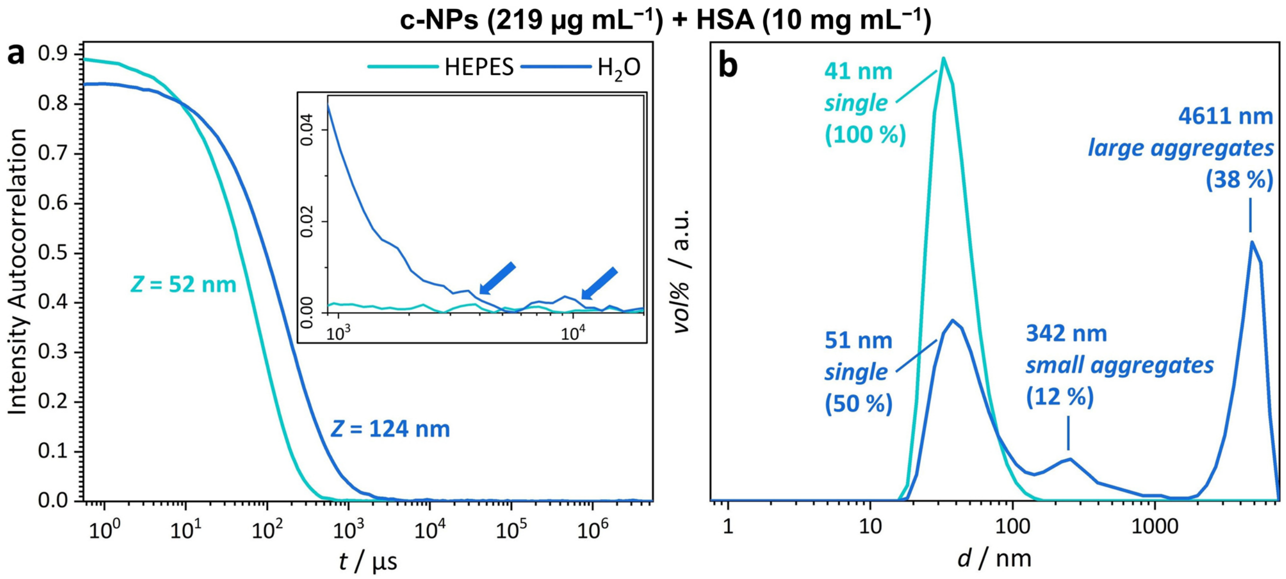

Figure S5 Exemplary (a) DLS correlograms and (b) size distribution profiles of samples with a monomodal (c-NPs in HEPES, turquoise) and a multimodal size distribution (c-NPs in H_2O, blue), respectively. Two representative DLS correlograms and the corresponding *Z*-values as well as the individual amount and particle sizes are given for c-NPs (219 µg mL^{-1}) with HSA (10 mg mL^{-1}) in HEPES (turquoise) and c-NPs (219 µg mL^{-1}) with HSA (10 mg mL^{-1}) in H_2O, each after two washing steps. The correlation function of c-NPs in H_2O exhibits two additional decays (marked with blue arrows in the inset), which indicates the presence of larger aggregates in the sample. The occurrence of aggregates in the aqueous sample can additionally be recognized in the elevated *Z*-value (Z_{H2O} = 124 nm) compared to the suspension in HEPES (Z_{Hepes} = 52 nm), where only single particles were present.

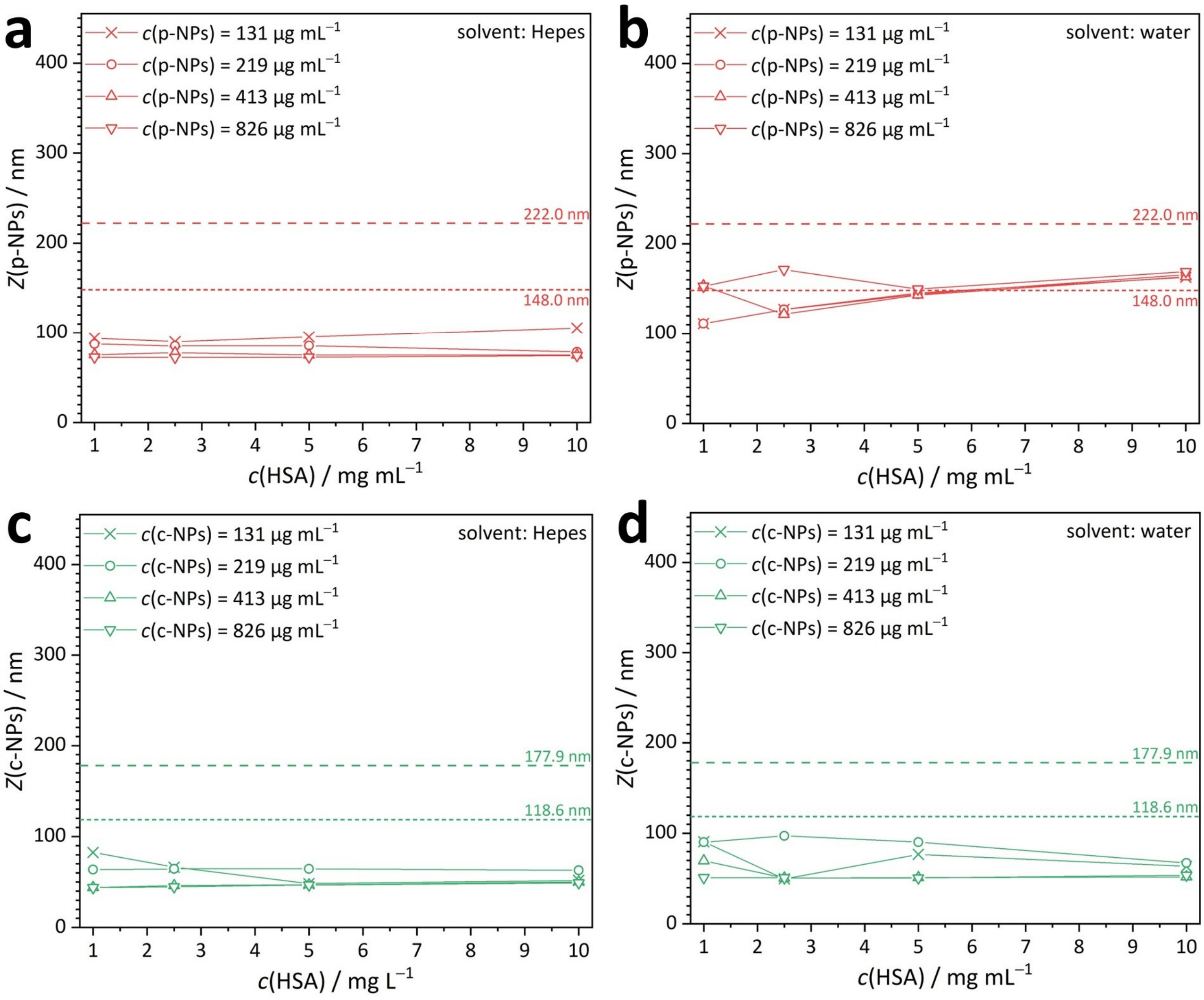

Figure S6 DLS results for commercial iron oxide nanoparticles in HEPES and water with addition of HSA in various concentrations. (a) p-NPs in HEPES showed stability for all analyzed HSA concentrations. (b) p-NPs were less stable in water and showed metastable behavior. (c) c-NPs could be stably dispersed in HEPES with various HSA contents. (d) Stability of c-NP suspensions in water was also measured for each analyzed HSA content.

Note to Figure S6:

To investigate the formation of a protein corona in more detail, HSA (up to 10 mg mL^{-1}) was added to p-NP or c-NP suspensions in ddH_2O, or HEPES (buffering capacity between pH 6.8 and 8.2). As described in the main text, the Z-mean extracted from DLS measurements was used to determine the stability of the NP suspensions (Figure S4). The protein human serum albumin (HSA, 66.5 kDa) has an isoelectric point of 4.7, i.e. the net charge of the molecule is negative at physiological pH. The nominal diameter in its globular structure is about 8 nm, but the protein has a tendency to dimerize or oligomerize under unfavorable conditions.[4]

Commercial p-NPs in HEPES were stable upon addition of HSA (1 to 10 mg mL^{-1}) regardless of the p-NP concentration (Z-means below $2d$ = 148.0 nm) (Figure S6a), and the particle size remained largely the same independent of the HSA concentration, pointing to a tightly bound surface protein corona, the so-called hard protein corona. Excess HSA may only loosely bind and may not be detected. By contrast, p-NPs were less stable in aqueous solutions with the addition of HSA (Figure S6b). Here, in some cases, conditions

were reached under which only metastable suspensions exist, for example at HSA concentrations of 10 mg mL^{-1}. In addition, in water an overall steady raise in particle size with increasing HSA content was observed, suggesting an extension of the soft protein corona either by unfolding of HSA or by association of further protein molecules.

In contrast, c-NPs were found to be stable in both, HEPES and water (Figure S6c/d) irrespective of the particle and HSA concentrations. We speculate that c-NPs mainly form a hard protein corona due to their highly negative zeta-potential ($\zeta_{\text{c-NPs}}$ = –111 mV, $\zeta_{\text{p-NPs}}$ = –52 mV; values before HSA addition).

Tables

Table S1 Iron oxide nanoparticle core sizes determined with imaging and scattering methods.

**Method**	Magnetosomes from *M. gryphiswaldense* (size in nm)	Fe_3O_4@citric acid (size in nm)	Fe_3O_4@phospholipid (size in nm)
**TEM**	40 ± 2	10 (core)<br>60 (cluster)	10 (core)<br>50 – 100 (cluster)
**SAXS**	32 ± 5 (core)<br>< 6 (shell)	8 ± 2 (core)	8 ± 2 (core)
**DLS**	83.5 ± 2.8	59.3 ± 7.2	74.0 ± 1.5

Table S2 Comparison of Z-means and size-values d (obtained from the main peak in the size distribution) for differently concentrated NPs in water. The values d_i and Z_i refer to single measurements, while d and Z are the mean values with standard deviation σ. The displayed results show that particle sizes are similar for all investigated NP concentrations. Note, that the d and Z-values are in a similar size range, demonstrating that the samples have one main NP component.

Fe_3O_4 NP species	c / µg mL^{-1}	d_i / nm	d / nm	σ / nm	c / µg mL^{-1}	Z_i / nm	Z / nm	σ / nm
M. gryphiswaldense magnetosomes (b-NPs)	27	82.0			27	82.0		
		84.4				78.8		
		80.1				77.7		
	131	86.3			131	85.4		
		83.6				79.6		
		87.4				80.9		
	219	82.9			219	92.2		
		78.4				82.5		
		86.0	**83.5** ±	**2.8**		82.5	**82.4** ±	**4.1**
Fe_3O_4@citric acid (c-NPs)	131	52.1			131	45.4		
		119.6*				45.3		
		66.4	**59.3** ±	**7.2**		56.8	**49.2** ±	**5.4**
Fe_3O_4@phospholipid (p-NPs)	131	75.7			131	62.7		
	219	74.3			219	60.6		
	413	72.0	**74.0** ±	**1.5**	413	59.4	**60.9** ±	**1.4**

Measurement data marked with * were not included in calculations of the mean value.

Table S3 DLS-derived particle sizes of iron oxide NPs in RPMI and DMEM, respectively.

**Medium**	***Z* (c-NPs) / nm**	***Z* (p-NPs) / nm**	***Z* (b-NPs) / nm**
RPMI	91	2884	812
DMEM	165	2543	1684

Table S4 Detailed composition of the used cell culture media.

Content	DMEM	RMPI
	concentration (mg L^{-1})	
**Inorganic salts**		
$Ca(NO_3)_2 \cdot 4\ H_2O$	-	100
$CaCl_2 \cdot 2\ H_2O$	265	-
$Fe(NO_3)_3$	0.1	-
$MgSO_4$	97.72	48.84
KCl	400	400
NaCl	6400	6000
$NaHCO_3$	-	2000
Na_2HPO_4	-	800
**Amino acids**		
glycine	30	-
L – arginine hydrochloride	84	200
L – asparagine	-	50
L – aspartate	-	20
L – cystine dihydrochloride	62.57	65
L – glutamine	584	300
L – glutamine acid	-	20
L – hystidine hydrochloride monhydrate	42	15
L – hydroxy proline	-	20
L – isoleucine	105	50
L – leucine	105	50
L – lysine hydrochloride	146	40
L – methionine	30	15
L – phenylalanine	66	15
L – proline	-	20
L – serine	42	30
L – threonine	95	20
L – tryptophane	16	5
L – tyrosine disodium salt	103.79	29
L – valine	94	20
**Vitamins**		
biotin	-	0.2
choline chloride	4	3
D – Ca – pantothenate	4	0.25
folic acid	4	1
nicotinamide	4	1
para-aminobenzoic acid	-	1
pyridoxale hydrochloride	4	1
riboflavine	0.4	0.2
thiamine hydrochloride	4	1
vitamin B12	-	0.005
i – inositol	7.2	35
**Further components**		
D – glucose	4500	2000
glutathione	-	1
phenol red	15.9	5
fetal bovine serum	2 – 19% (v/v)	

Table S5 Particle sizes of iron oxide NP suspensions in different media as determined by DLS.

**Medium**	***c***	***Z* (c-NPs) / nm**		***Z* (p-NPs) / nm**	
		c = 131 µg mL^{-1}	c = 219 µg mL^{-1}	c = 131 µg mL^{-1}	c = 219 µg mL^{-1}
NaCl	25 mmol L^{-1}	1479.0	327.5	113.2	94.9
	75	87.7	176.8	93.0	86.9
	150	56.4	242.9	112.2	148.0
	200	582.5	320.4	483.2	182.3
	300	1099.0	1202.0	1241.0	1265.0
	500	828.0	998.1	1027.0	1197.0
$MgSO_4$	0.1 mmol L^{-1}			59.2	57.3
	0.25			58.8	58.4
	0.5	40.9	73.7	79.7	77.5
	1.0	42.9	46.4	261.4	436.4
Inositol	5 mg L^{-1}	44.0	42.6	58.5	82.7
	10	43.7	43.1	110.8	56.7
	20	51.9	42.2	67.6	97.1
	35	51.6	42.6	60.1	56.7
	50	48.4	41.4	113.8	97.5

Table S6 Composition of the gels and buffers used for SDS-PAGE.

**Component**	**Quantity**		
	Stacking gel	Running gel	
Acrylamide	5%	8%	22.5%
Rotiphorese Gel 30 (acrylamide-bisacrylamide mixture)	2.0 mL	5.6 mL	18.0 mL
Stacking gel buffer 0.5 M Tris[a], 0.4% SDS[b] (w/v), pH 6.8	-	6.0 mL	6.0 mL
Running gel buffer 3 M Tris[a], 0.4% SDS[b] (w/v), pH 8.85	4.6625 mL	-	-
ddH_2O	12.0 mL	12.4 mL	6.0 mL
APS[c] (10% w/v)	63.75 mL	80 µL	80 µL
TEMED[d]	18.75 µL	12 µL	12 µL

**Electrophoresis buffer**
0.05 M Tris[a]<br>0.19 M glycine<br>0.1% SDS (w/v)<br>pH 8.6 - 8.7

[a] tris(hydroxymethyl)aminomethane

[b] sodium dodecyl sulfate

[c] ammonium persulfate

[d] *N,N,N',N'*-tetramethylethylenediamine

Table S7 Product information of commercial iron oxide nanoparticles as specified by the manufacturers.

	**p-NPs**[5]	**c-NPs**[6]
**producer**	micromod Partikeltechnologie GmbH	chemicell GmbH
**product name**	iron oxide particles	fluidMAG-CT
**product number**	45-111-701	4122-5
**surface functionalization**	phospholipid 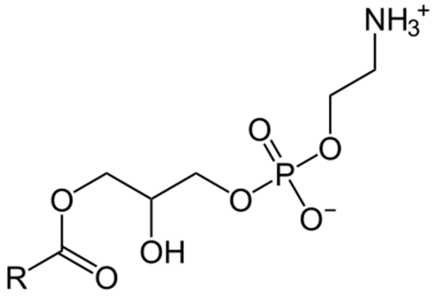	citric acid (sodium salt) 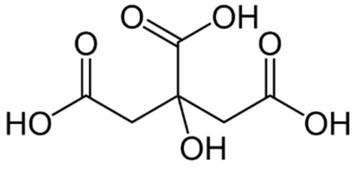
**core**	iron oxide	magnetite
**NP concentration**	10 mg mL^{-1}	25 mg mL^{-1}
**dispergent**	water	water
**hydrodynamic diameter**	70 nm	50 nm
**form**	clustered	clustered

References Supporting Information

(1) Technical Committee ISO/TC 24/SC 4 *ISO 22412: Particle Size Analysis - Dynamic Light Scattering (DLS)*, 2nd ed.; International Organization for Standardization, 2017.

(2) The SasView Project Mass Fractal Model https://www.sasview.org/docs/user/models/mass_fractal.html (accessed 2023 -04 -21).

(3) The SasView Project Sphere Model https://www.sasview.org/docs/user/models/sphere.html (accessed 2023 -04 -21).

(4) Carter, D. C.; Ho, J. X. *Adv. Protein Chem.* **1994**, *45*, 153–176.

(5) micromod Partikeltechnologie GmbH iron oxide particles 45-111-701 www.micromod.de/wp-content/uploads/datasheets/45-111-701_tds_de.pdf (accessed 2022 -09 -19).

(6) Chemicell GmbH fluidMAG-CT 4122-5 www.chemicell.com/products/nanoparticles/docs/PI_fluidMAG-CT_4122.pdf (accessed 2022 -09 -19).
